# Inhibitory Effect of Polydatin Against *Aeromonas hydrophila* Infections by Reducing Aerolysin Production

**DOI:** 10.3389/fvets.2022.937463

**Published:** 2022-07-14

**Authors:** Jing Dong, Tianhui Yan, Qiuhong Yang, Shun Zhou, Yi Song, Yongtao Liu, Liang Ma, Ning Xu, Yibin Yang, Xiaohui Ai

**Affiliations:** ^1^Yangtze River Fisheries Research Institute, Chinese Academy of Fishery Sciences, Wuhan, China; ^2^College of Food Science and Engineering, Bohai University, Jinzhou, China; ^3^Chinese Academy of Fishery Sciences, Beijing, China

**Keywords:** polydatin, aerolysin, hemolytic activity, anti-virulence, *Aeromonas hydrophila*

## Abstract

The fast-growing demand for aquatic products has led to the rapid development of aquaculture. However, diseases caused by bacterial pathogens result in severe economic losses all over the world. Although the introduction of antibiotics to aquaculture decreased the mortality of infectious diseases, the emergence of antibiotic resistance caused treatment failure. Therefore, drugs with novel strategies are needed for combatting infections caused by resistant bacterial strains. In the present study, aerolysin was identified as a target for developing drugs from natural compounds against *Aeromonas hydrophila* (*A. hydrophila*) infections. We found that polydatin without an inhibitory effect against *A. hydrophila* growth could decrease the hemolysis mediated by aerolysin. In both western blot and qPCR assays, the addition of polydatin decreased the production of aerolysin by downregulating the aerolysin encoding gene. Moreover, cell viability and animal studies found that polydatin could reduce the pathogenesis of *A. hydrophila* both *in vitro* and *in vivo*. Taken together, these findings provided a novel approach and candidate for treating resistant *A. hydrophila* infections in aquaculture.

## Introduction

Teleost fish have become one of the largest protein sources for humans by the extensive development of aquaculture (1). According to the report of the Food and Agriculture Organization, aquatic production from cultured fish was one of the fastest-growing departments, which offered about 179 million tons of production in 2018 (2, 3). However, challenges caused by bacterial infections threaten the sustainable development of the industry, which bring a global estimate of economic losses of several billion US$ per year (4). Therefore, controlling bacterial infections is essential for the healthy development of the industry. Antibiotics are the mainstay in attempts to control bacterial infections in aquaculture. However, the emergence and spread of antibiotic resistance have been observed (4). Moreover, the increasing use of antibiotics results in antibiotic residues and environmental risks (5). Thus, alternatives to antibiotics are needed to control bacterial infections in aquaculture.

*Aeromonas hydrophila* (*A. hydrophila*) is a Gram-negative bacterium widely distributed in aquatic environments and is responsible for a range of diseases in cultured fish (6). Moreover, *A. hydrophila* can be isolated from a variety of foods, such as meat, milk, dairy products, and even vegetables (7). Therefore, *A. hydrophila* has been recognized as a foodborne pathogen because it can be transmitted from diseased fish, contaminated water, and uncooked seafood to human (6). Virulence factors secreted by pathogenic bacteria are involved in the invasion of host cells, evasion mechanisms, and nutrient acquisition (8). The virulence factors of *A. hydrophila* are aerolysin, hemolysin, and adhesins (9). Aerolysin, a pore-forming toxin, plays a critical role in the pathogenicity of *A. hydrophila*, which is secreted as an inactive water-soluble dimer named proaerolysin (10). Proaerolysin can be activated by cleaving a flexible 43-residue loop nearing the C-terminus (11). Aerolysin can form a channel pore on the surface of target cells and results in cell death (12). A number of mammalian cells are sensitive to the toxin. The critical role of aerolysin has been clarified by the protective effect of immunization against the toxin and decreased pathogenesis of knocking out of aerolysin encoding gene (13). Therefore, aerolysin is an ideal target identifying novel drugs against *A. hydrophila* infections.

**Figure 1:**
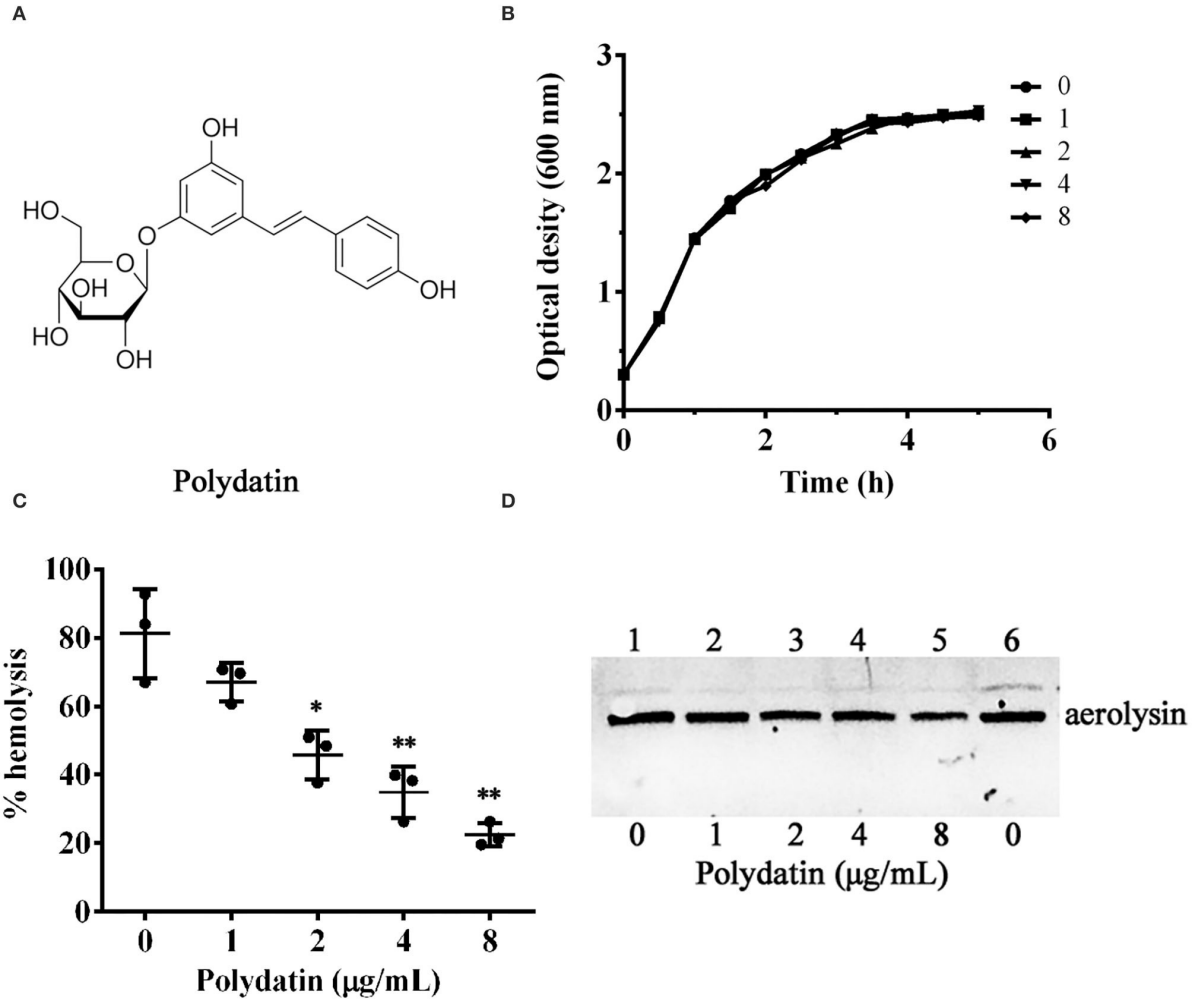
Inhibitory effect of polydatin against aerolysin mediated hemolysis. **(A)** The chemical structure of polydatin. **(B)** Influence of different concentrations of polydatin on bacterial growth. **(C)** Reduction of the hemolytic activity of bacterial supernatants co-cultured with indicated concentrations of polydatin. **(D)** Impact of polydatin on aerolysin production in bacterial supernatants determined by western blot. Data shown in **(C)** are the mean ± SD of three independent assays, * represented *p* < 0.05, while ** was *p* < 0.01.

Herbal medicines have been widely used for their strong antimicrobial effects and primary healthcare benefits in most of the world's populations (14). Therefore, herbal medicines and their constituents are considered as alternatives and sources of antibiotics (15). Polydatin (3,4′,5-trihydroxystilbene-3-O-β-D-glucoside, Figure 1A) is a natural compound belonging to stilbenes that can be derived from *Polygonum cuspidatum* Sieb. et Zucc (16, 17). Polydatin exhibits a variety of biological activities, such as cardiovascular protection, neuroprotection, anti-inflammation, immune-regulation, anti-oxidation, and anti-tumor activities (18). However, there is little knowledge about its effect on *A. hydrophila*. In the present study, we found that polydatin could inhibit the pathogenesis of *A. hydrophila* by inhibiting the production of aerolysin. The findings provided a novel approach in dealing with *A. hydrophila* infections in aquaculture.

## Materials and Methods

### Bacterial Strains and Regents

*Aeromonas hydrophila* XS-91-4-1 was isolated from diseased *Hypophthalmichthys molitrix* from Prof. Aihua Li at the Institute of Hydrobiology, Chinese Academy of Sciences. *Escherichia coli* (*E. coli*) ATCC 25922 was purchased from ATCC and stored in our laboratory. Polydatin (CAS No. 27208-8006) with a purity of 98% was a commercial product of Sichuan Weikeqi biological technology Co., LTD, while enrofloxacin (CAS No. 93106-60-6) with a purity of 99% was purchased from the National Institutes for Food and Drug Control (Beijing, China). For *in vitro* studies, polydatin was prepared by dimethyl sulfoxide (DMSO, Sigma-Aldrich, St. Louis, USA) at 40.96 mg/ml. For the *in vivo* study, polydatin was dissolved in 0.5% carboxymethylcellulose sodium at 20 mg/ml.

### Measurement of Minimal Inhibitory Concentrations (MICs)

Minimal inhibitory concentrations (MICs) of *A. hydrophila* XS-91-4-1 to enrofloxacin and polydatin were determined by the broth serial-dilution method in 96-well plates according to the guidance of CLSI M45-A3 (19). In brief, enrofloxacin and polydatin were diluted by two-fold in volumes of 100 μl in each well by Mueller-Hinton Broth (MHB, Hopebiol, Qingdao, China) at concentrations ranging from 64 to 0.125 μg/ml and 512 to 1 μg/ml, respectively. Bacterial strains stored at −70°C were thawed and sub-cultured for Luria-Bertani (LB, Hopebiol, Qingdao, China) medium at 28°C for 16–18 h. Bacterial cells were harvested by centrifugation (8,000 g, 2 min, 4°C) and washed three times with sterile PBS. Then, the concentration of bacterial cells was adjusted to about 5 × 10^5^ CFU/ml using the MHB medium by McFarLand standards. Bacterial suspension at a volume of 100 μl was added to each well and further incubated at 35°C for 16–20 h. DMSO was added into drug free well and was served as a positive control. *E. coli* ATCC 25922 was used as a quality control strain. The lowest concentration without visible bacterial growth was defined as the MICs.

### Growth Curves Assay

*Aeromonas hydrophila* XS-91-4-1 was cultured in the LB medium for 16–18 h, then 1 ml of bacterial culture was inoculated to 100 ml fresh LB medium and further incubated at 28°C until the optical density at 600 nm (OD_600nm_) reached 0.3. Bacterial cultures were divided into 5 glass flasks at a volume of 10 ml and then indicated concentrations of polydatin were added to each flask. The mixtures were further cultured for 5 h and the growth of bacteria was monitored by determining the absorption at 600 nm every 30 min. A drug-free group was co-cultured with DMSO to determine the impact of DMSO on bacterial growth.

### Hemolysis Assay of Bacterial Supernatants

An overnight bacterial culture (1 ml) was added to 100 ml of fresh LB medium and further cultured to OD_600nm_ of 0.3 at 28°C. Bacterial suspension was divided into five 50-ml glass flasks at a volume of 10 ml, then polydatin at concentrations ranging from 0 to 8 μg/ml was added to each flask and further incubated by shaking up to reach an OD_600nm_ of 1.5. Bacterial supernatants were collected by centrifugation and were used for determining aerolysin-induced hemolysis. Supernatants were treated with trypsin at room temperature for 10 min to activate aerolysin after being sterilized by 0.22 μm filters. Activated supernatants were used for the hemolytic activity assay. Briefly, the hemolytic reaction system was brought up in 1.5 ml tubes by 875 μl reaction buffer (25 mM Tris, 150 mM NaCl, pH = 7.2), 100 μl bacterial supernatant, and 25 μl freshly washed sheep red blood cells. The systems were mixed gently and further incubated at 37°C for 20 min. The mixtures were centrifuged at 10,000 g for 2 min to remove unlyzed erythrocytes. Erythrocytes treated with 0.1% Triton X-100 was served as a positive control (100% hemolytic activity). The hemolytic activities of bacterial supernatants treated with indicated concentrations of polydatin were determined by measuring the absorptions at 543 nm.

### Western-Blot Assay

A primary anti-aerolysin polyclonal antibody was prepared by New Zealand white rabbits in accordance with the Animal Welfare and Research Ethics Committee of Yangtze River Fisheries Research Institute. Aerolysin was overexpressed and purified as per our previously reported method (20). Rabbits were hypodermically injected with aerolysin (500 μg) plus complete Freund's adjuvant, then boosted two times with aerolysin plus incomplete Freund's adjuvant at 2-weeks intervals. Serum collected from the carotid artery was used for the western blot assay.

Samples were prepared as described in the hemolysis assay. Before sampling, total proteins in supernatants were determined using a Pierce^TM^ BCA protein assay kit (Thermo Fisher Scientific, MA, USA). Samples were loaded on a sodium dodecyl sulfate (SDS)-polyacrylamide (12%) gel after being mixed with Laemmli sampling buffer and boiled. Proteins in the gel were transferred to a 0.22 μm PVDF membrane by a semi-dry transfer cell. Then, the membrane was incubated with the primary anti-aerolysin polyclonal antibody at a concentration of 1:1,000 for 2 h after being blocked by 5% non-fat milk in TBST for 1 h. The membrane was washed with TBST for 10 min, three times, and further incubated with a HRP-conjugated secondary goat anti-rabbit antiserum at a concentration of 1:40,000 for 1 h. After washing, proteins in the membrane were detected by ECL Western blotting detection regents. Pictures were photographed by a ChemiDoc XRS+ imager.

### Real-Time PCR

Pretreated *A. hydrophila* XS-91-4-1 cultures as mentioned earlier in the hemolysis assay were used for RNA extraction. Bacterial cells were acquired by centrifugation for 10 min at 4°C when OD600 nm of the cultures reached 1.5. A MolPure bacterial RNA kit was used to isolate total RNA from bacterial cells. cDNA was synthesized after determining the concentration of total RNA. Then a real-time PCR assay was conducted to determine the effect of polydatin on the transcription of aerolysin encoding gene *aerA*. As an internal housekeeping control gene, 16s rRNA was employed. Primer pairs for detecting *aerA* gene were 5'- TCTACCACCACCTCCCTGTC-3'(forward) and 5- GACGAAGGTGTGGTTCCAGT-3'(reverse), while 16s rRNA were 5'- TAATACCGCATACGCCCTAC-3'(forward) and 5- ACCGTGTCTCAGTTCCAGTG-3'(reverse) (21). CT values were used to calculate the relative expression levels of *aerA* by 2^−ΔΔT^ method as described in a previous study (22).

### Cell Viability Assay

A549 alveolar carcinoma cells were cultured in the Dulbecco's Modified Eagle Medium (DMEM, Thermo Fisher Scientific, MA, USA) plus 10% fetal bovine serum with 5% CO_2_ at 37°C. Cells were seeded into a 96-well plate at a density of 1.5 × 10^5^ cells per well and further cultured overnight at 37°C. To determine the protective effect of polydatin against aerolysin-induced cell injury, lactate dehydrogenase (LDH) release and live/dead assays were performed. Cells were incubated with bacterial supernatants treated with the indicated concentrations of polydatin for 1 h at 37°C. Cell supernatants were obtained and 120 μl of which were moved to a new plate, then 60 μl of LDH working solution was added to each well and further incubated at room temperature for 30 min. The percentage of LDH release was determined by measuring the OD_490nm_ values using a multi-mode microplate reader. For live/dead assay, cells treated previously were washed and were incubated with Calcein AM and Propidium iodide at concentrations of 2 and 4.5 μM, respectively. After a 30 min staining, cells were imaged by a fluorescence microscope.

### *In vivo* Studies

Animal studies were carried out according to the guidance of the Institutional Animal Welfare and Research Ethics Committee. Channel catfish weighing 200 ± 10 g were separated into three groups, containing 30 fish in each group, and were maintained in 100 L glass tanks for 7 days prior infection. Bacterial cells of *A. hydrophila* XS-91-4-1 were collected when cultured to mid-logarithmic phase in LB medium. After washing, bacterial cells were re-suspended to a density of about 1.5 × 10^8^ CFU/ml in sterile PBS. Then, a fish infection model was established by intraperitoneal injection of bacterial suspension at a volume of 200 μl, while fish in the negative group were injected with the same volume of sterile PBS. A gavage needle was used to administer polydatin to fish in the polydatin treating group at a dosage of 20 mg/kg 6 h post-infection and then 12-h intervals for 3 days. Fish in positive and negative groups were given 0.5% carboxymethylcellulose as control. Deaths were monitored every 24 h for 8 days and mortality was analyzed.

### Statistical Analysis

The normality of hemolytic activity, gene expression, and cell viability data was analyzed by one sample K-S test and explore test, then the statistical significance was determined by Student's *t*-test. Kaplan–Meier estimates and log-rank tests were used to analyze the survival rate of animal studies by GraphPad Prism v5.04.

## Results

### Influence of Polydatin on *A. hydrophila* Growth

The MICs of enrofloxacin and polydatin against *A. hydrophila* XS-91-4-1 were 4 μg/ml and 512 μg/mL, indicating that polydatin had little inhibitory effect against *A. hydrophila*. Moreover, growth curves at 5 h were determined to assess the effect of polydatin on bacterial growth. As shown in Figure 1B, no impact was observed when *A. hydrophila* co-cultured with polydatin at concentrations ranging from 1 to 8 μg/ml. The addition of DSMO to polydatin-free groups both in MIC determination and growth curves assay showed that DMSO had no role on bacterial growth.

### Inhibitory Effect of Polydatin on Hemolysis of *A. hydrophila* and Aerolysin Production

A hemolytic activity assay was performed to determine the impact of polydatin on hemolysis of bacterial supernatants co-cultured with indicated concentrations of polydatin. As shown in Figure 1C, hemolysis was reduced at a dose-dependent manner. The hemolytic activity decreased to 22.46 ± 3.42% when co-cultured with 8 μg/ml polydatin compared with 81.24 ± 13.08% of drug-free group. The hemolysis of bacterial supernatants with polydatin of 2 μg/ml was remarkably reduced (*p* = 0.024) compared with the polydatin-free group. Moreover, the western blot assay was performed to analyze the inhibitory effect of polydatin on the production of aerolysin in bacterial supernatants. As shown in Figure 1D, the levels of aerolysin in supernatants were decreased at a dose-dependent manner.

### Polydatin Inhibited the Transcription of *aerA* Gene

**Figure 2:**
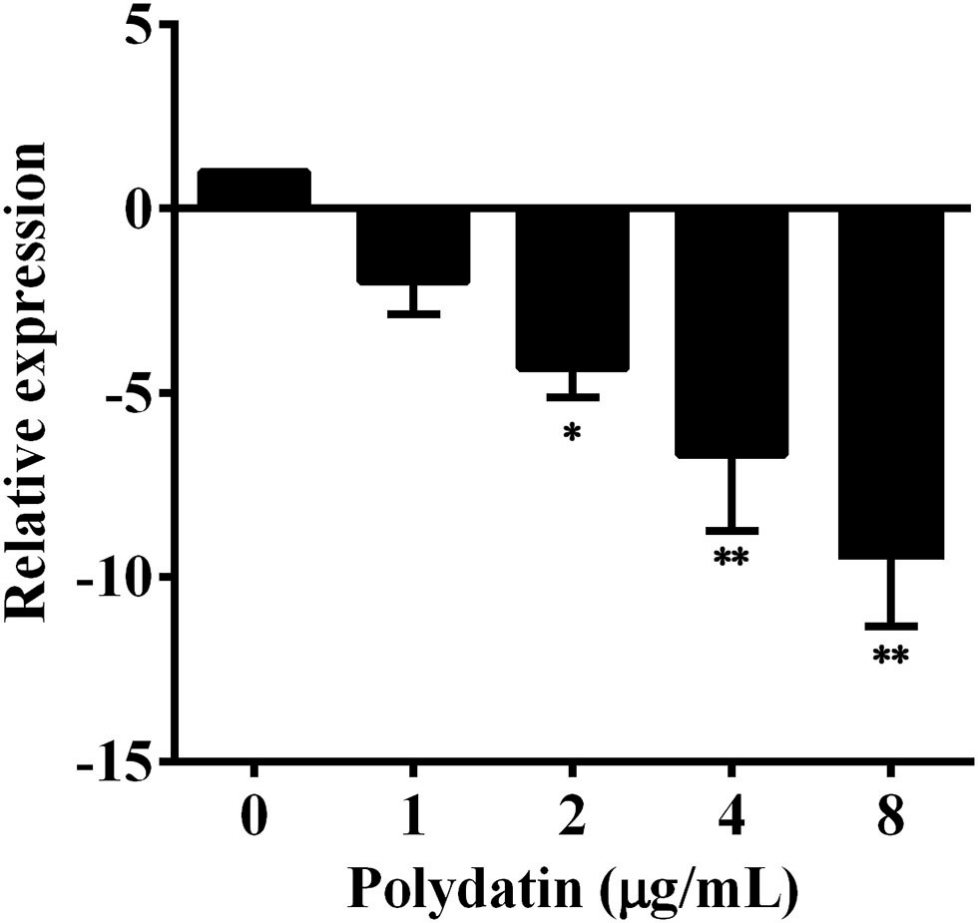
Effects of polydatin on the expression levels of the *aerA* gene. Data are represented as the mean ± SD of three independent assays, * indicates *p* < 0.05, ** indicates *p* < 0.01.

Real-time PCR (RT-PCR) was conducted to determine the effect of polydatin treatment on *aerA* transcription. As shown in Figure 2, the transcription of *aerA* was decreased following the addition of polydatin. The *aerA* gene was downregulated 1.99, 4.33, 6.68, and 9.51-fold with polydatin at concentrations ranging from 1 to 8 μg/ml compared with polydatin-free group. When *A. hydrophila* XS-91-4-1 co-cultured with polydatin at 2 μg/ml, the transcription of *aerA* was significantly (*p* = 0.018) downregulated. The findings demonstrated that polydatin inhibited the hemolytic activity and aerolysin production by suppressing the transcription of *aerA* gene.

### Polydatin Protected A549 Cells From Aerolysin Mediated Cell Injury

**Figure 3:**
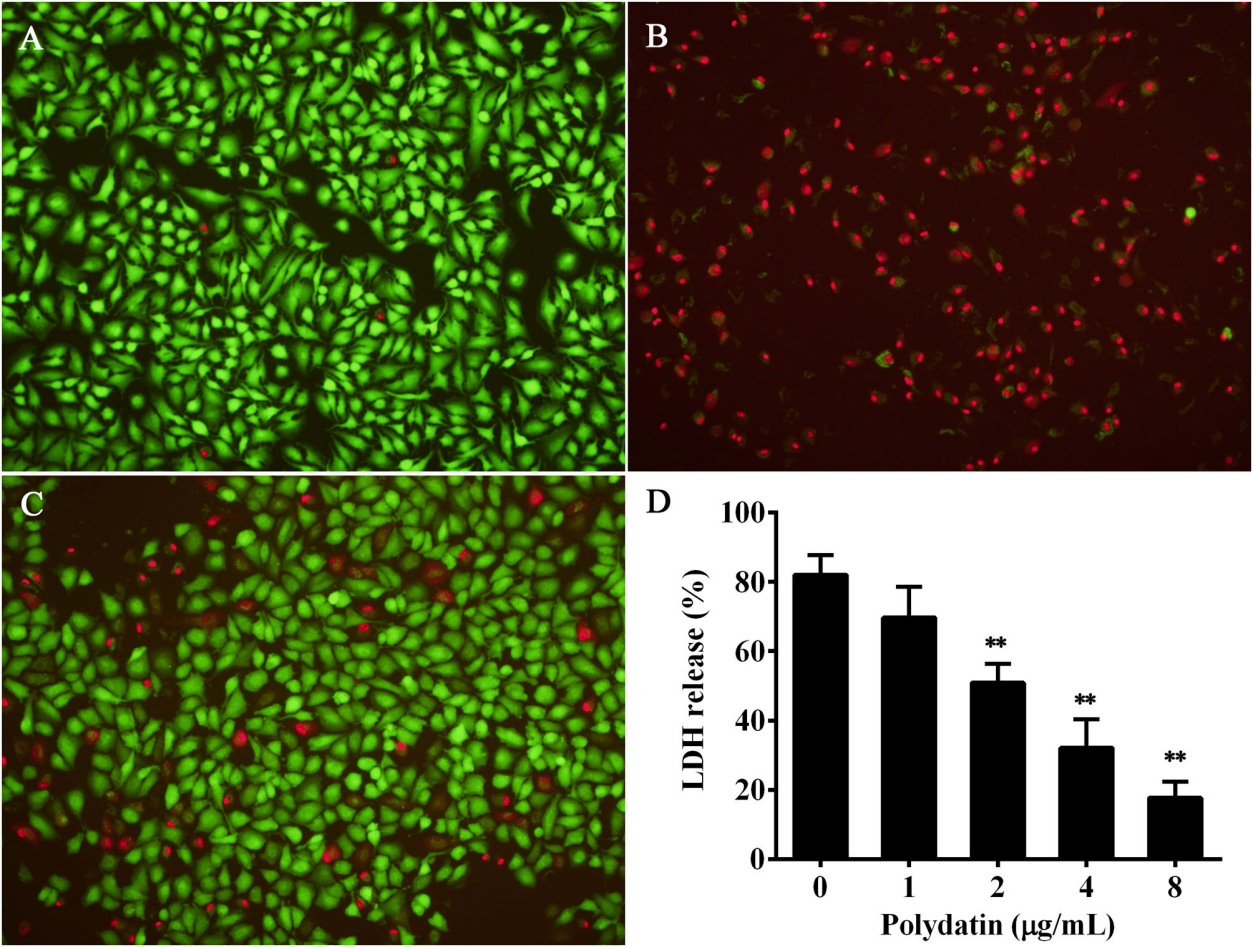
Polydatin protected A549 cells against aerolysin mediated cell injury. **(A–C)** were cell viability determined by live/dead staining, **(A)** live cells without any treatment, **(B)** cells co-cultured with bacterial supernatant without polydatin treatment, **(C)** cells co-cultured with bacterial supernatant treated with 8 μg/ml polydatin. **(D)** Cell injury determined by lactate dehydrogenase (LDH) release assay. Data in **(D)** are the mean ± SD of three independent assays, ** indicates *p* < 0.01.

It has been proven that A549 cells can be used as a cell line by assessing the cytotoxicity induced by aerolysin (23). Therefore, the protective effect of polydatin against aerolysin mediated cell injury was determined using A549 cells by live/dead staining and LDH release assays. Cells without any treatment were live and were stained with green fluorescence (Figure 3A), while the red of cells treated with polydatin-free bacterial supernatant (Figure 3B). Cells co-cultured with bacterial supernatant plus 8 μg/ml polydatin showed a visible decrease in dead cells (Figure 3C). Moreover, the results of the LDH release assay showed LDH release was decreased with increasing concentrations of polydatin (Figure 3D). When polydatin reached to 2 μg/ml, LDH release of A549 cells was reduced to 50.89 ± 5.42% compared with 82.03 ± 5.73%, which was statistical significance (*p* = 0.002, Figure 3D). Taken together, these findings demonstrated that treatment with polydatin could provide a significant protection to A549 cells from aerolysin induced cell injury.

### Polydatin Protected Channel Catfish Against *A. hydrophila* Infection

**Figure 4:**
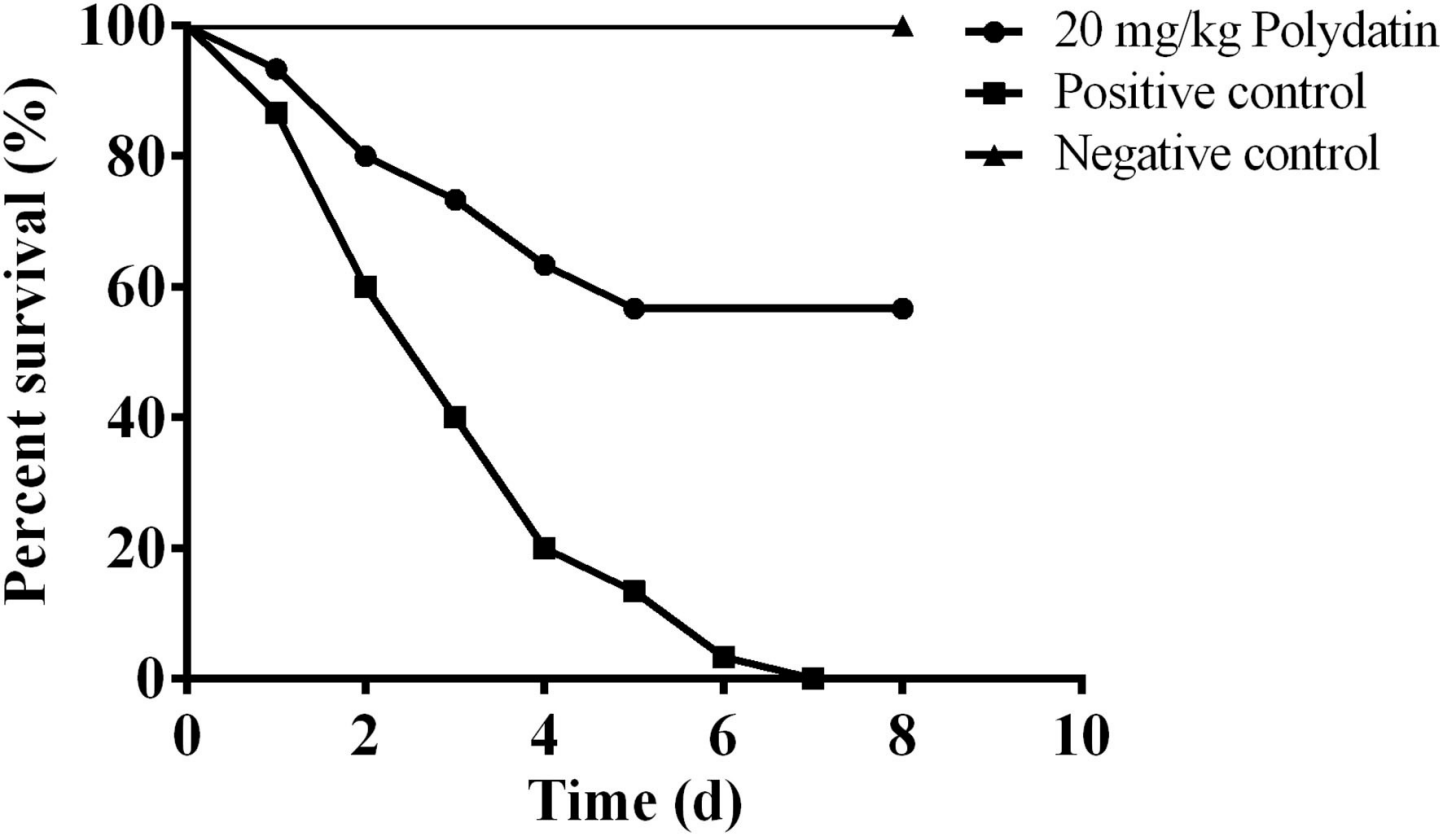
The survival percentage of channel catfish challenged with *Aeromonas hydrophila (A. hydrophila)* and treated with or without polydatin. The survival rates of each group were analyzed by log-rank test, and the statistical significance (*p* = 0.0,003) was observed.

*In vitro* results demonstrated that polydatin could significantly reduce aerolysin-mediated cell injury. Moreover, a previous study found that *A. hydrophila* deleting *aerA* showed a significant decrease in pathogenesis (24). Therefore, it is reasonable to believe that polydatin might have a therapeutic effect against *A. hydrophila* infection. As shown in Figure 4, deaths were observed on the first day post-infection, the survival rate of fish in the positive control group in 8 days' course was 0%, while 56.67% of fish in the 20 mg/kg polydatin treated group, which showed statistical significance (*p* = 0.0,003). No death was observed in the negative control group during the experimental period indicating that *A. hydrophila* infection resulted in deaths in the positive control and polydatin treated groups. These results suggested that the inhibition of aerolysin by polydatin could provide a significant protection to channel catfish challenged with *A. hydrophila*.

## Discussion

The discovery of antibiotics has changed the situation of infectious diseases caused by bacterial pathogens and saved human lives (25). However, antibiotic resistance has been observed because of the overuse and selective pressure by antibiotics. *A. hydrophila* is one of the most important bacterial pathogens in freshwater fisheries, which has been reported to be resistant to a number of antibiotics (26). In the present study, we found that *A. hydrophila* XS-91-4-1 was resistant to enrofloxacin according to CLSI M45-A3 (19). Our previous study demonstrated that fish challenged with *A. hydrophila* XS-91-4-1 and administered with enrofloxacin resulted in more severe mortality compared with fish infected with bacteria only, which revealed that enrofloxacin could not be used for treating infections caused by resistant bacterial strains. Therefore, novel anti-bacterial strategies were urgently needed for battling infections caused by resistance bacteria. Aerolysin, belonging to the pore forming toxin family, has been proved as the chief virulence factor of pathogenic *A. hydrophila* (24). Thus, aerolysin was selected as a target for identifying drugs against the pathogenesis of *A. hydrophila* from natural compounds isolated from traditional Chinese herbal medicines (27).

A number of biomedical properties of polydatin have been identified, but the anti-bacterial effect of polydatin was little reported. Cao et al. investigated the inhibitory effects of components isolated from *Rhizoma Polygoni Cuspidati* against methicillin-resistant *Staphylococcus aureus* (MRSA). The results showed that MIC of polydatin against MRSA 252 was higher than 512 μg/ml indicating that polydatin could hardly inhibit the growth of MRSA *in vitro* (28). However, the inhibitory effect of polydatin against aquatic pathogens was not found. Similar to the study reported by Cao et al., our present study showed that the MIC of polydatin against *A. hydrophila* was 512 μg/ml. However, we found that polydatin could reduce the hemolysis mediated by aerolysin at much lower concentrations than MIC, indicating that polydatin would give less selective pressure to *A. hydrophila* than antibiotics. Although antimicrobial resistance is ancient and is the results of the interaction between bacteria and their environment, selective pressures provided by antibiotics accelerates the process of resistance (29). Therefore, polydatin provided less selective pressure, might decrease the risk of inducing resistance. Inhibition of the production and activity of aerolysin can decrease the hemolytic activity of the enzyme. Thus, the inhibition of polydatin against purified aerolysin was performed, but no inhibitory effect was observed. Moreover, western blot assay showed that aerolysin in bacterial supernatants was decreased following the increasing concentrations of polydatin in co-culture systems.

Chen et al. found that neuraminidase of influenza A influenza virus could be inhibited by polydatin with an IC_50_ of 129.8 μM, indicating that polydatin was significant to drug discovery against influenza (30). Xu et al. reported that polydatin had the activity against the severe acute respiratory syndrome coronavirus 2 (SARS-CoV-2) by inhibiting the proteolytic activity of 3CLpro and Plpro (31). These findings reveal that polydatin has the potential to be developed as drugs against infectious diseases. However, polydatin as anti-virulence agents against bacterial pathogens has not been reported. Polydatin was the first time identified that had anti*-hydrophila* effect *via* inhibiting the expression of aerolysin. Polydatin is a glucoside of resveratrol (3,4′,5-trihydroxystilbene). Moreover, our previous study has demonstrated that resveratrol is an inhibitor of quorum sensing of *A. hydrophila*, which could provide a protection to channel catfish challenged with *A. hydrophila* (23). The inhibitory effect against the quorum sensing of polydatin was determined by biofilm formation and qPCR. However, polydatin had no role on biofilm formation and transcription of quorum sensing related genes. Although the modes of action of polydatin and resveratrol were different, similar effects against *A. hydrophila* infection in a channel catfish model were achieved. The root of *Polygonum cuspidatum* Sieb. et Zucc, major source of polydatin, is one of the main components of Huhuang Heji which is used for treating *A. hydrophila* infections in aquaculture in China. Thus, the discoveries in the present study not only provided a promising candidate against *A. hydrophila* infections but partly clarified the mechanism of traditional Chinese medicine in treating bacterial infections.

## Data Availability Statement

The original contributions presented in the study are included in the article/supplementary material, further inquiries can be directed to the corresponding author/s.

## Ethics Statement

The animal study was reviewed and approved by Animal Welfare and Research Ethics Committee of Yangtze River Fisheries Research Institute.

## Author Contributions

JD and XA designed the experiments. JD, TY, and LM carried out the experiments. JD, YY, and TY collected data. JD and NX wrote this manuscript. YL, YS, and SZ checked and revised the manuscript. QY, JD, and SZ contributed to data analysis. All the authors discussed the results and commented on the manuscript.

## Funding

This work was supported by the National Key R&D Program of China (No. 2019YFD0900104) and China Agriculture Research System of MOF and MARA (CARS-46).

## Conflict of Interest

The authors declare that the research was conducted in the absence of any commercial or financial relationships that could be construed as a potential conflict of interest.

## Publisher's Note

All claims expressed in this article are solely those of the authors and do not necessarily represent those of their affiliated organizations, or those of the publisher, the editors and the reviewers. Any product that may be evaluated in this article, or claim that may be made by its manufacturer, is not guaranteed or endorsed by the publisher.
